# Impact of enriched environment on motor performance and learning in mice

**DOI:** 10.1038/s41598-024-56568-3

**Published:** 2024-03-12

**Authors:** S. Dijkhuizen, L. M. C. Van Ginneken, A. H. C. IJpelaar, S. K. E. Koekkoek, C. I. De Zeeuw, H. J. Boele

**Affiliations:** 1 Department of Neuroscience, Erasmus MC, 3015 GD Rotterdam, The Netherlands; 2 Netherlands Institute for Neuroscience, Royal Academy of Arts and Sciences (KNAW), 1105 BA Amsterdam, The Netherlands; 3 Princeton Neuroscience Institute, Princeton, NJ 08540 USA

**Keywords:** Physical enrichment, Social enrichment, Cage enrichment, Delay eyeblink conditioning, Rotarod, ErasmusLadder, Mice, Neuroscience, Learning and memory, Motor control

## Abstract

Neuroscience heavily relies on animal welfare in laboratory rodents as it can significantly affect brain development, cognitive function and memory formation. Unfortunately, laboratory animals are often raised in artificial environments devoid of physical and social stimuli, potentially leading to biased outcomes in behavioural assays. To assess this effect, we examined the impact of social and physical cage enrichment on various forms of motor coordination. Our findings indicate that while enriched-housed animals did not exhibit faster learning in eyeblink conditioning, the peak timing of their conditioned responses was slightly, but significantly, improved. Additionally, enriched-housed animals outperformed animals that were housed in standard conditions in the accelerating rotarod and ErasmusLadder test. In contrast, we found no significant effect of enrichment on the balance beam and grip strength test. Overall, our data suggest that an enriched environment can improve motor performance and motor learning under challenging and/or novel circumstances, possibly reflecting an altered state of anxiety.

## Introduction

Environmental deprivation imposes serious constraints on brain development, cognitive functioning, memory formation, motor performance, social interaction, and even body growth^1–4^. It is therefore not a surprise that individually housed rodents, used in biomedical research, often show impairments in all of these domains^4–8^. To remedy this problem, Donald Hebb in 1947 introduced the concept of *environmental enrichment*, involving rats growing up as pets and mostly spending their time outside their cage compared to standard-housed rats^9^. Numerous studies now have shown, in both rats and mice, that this enriched physical and social rearing results in improved brain development and cognitive functioning. For instance, cage enrichment leads to enhanced neural plasticity in the hippocampus, leading to improved memory formation in learning tasks like Morris water maze and contextual fear conditioning^6,10–27^. Similarly, cage enrichment induces molecular and neural structural changes in prefrontal cortex^28^, amygdala^16^ and locus coeruleus^29^.

In the *cerebellum*, it has been shown that physical enrichment causes long-term metabolic plasticity in the form of increased cytochrome oxidase activity^30^, which is accompanied by angiogenesis^31,32^. These changes were seen in spino-cerebellum, but not in the hemispheres crus 1 and 2. In addition, in the cerebellar nuclei, enriched housing results in a decreased expression of perineural nets, the extracellular matrix proteins that are important for stabilization of neural connections^33^. Since the cerebellum is a crucial brain structure for regulation of motor control, potential effects of environmental enrichment on motor learning and motor performance may be revealed in various cerebellum-dependent tasks.

**Figure 1:**
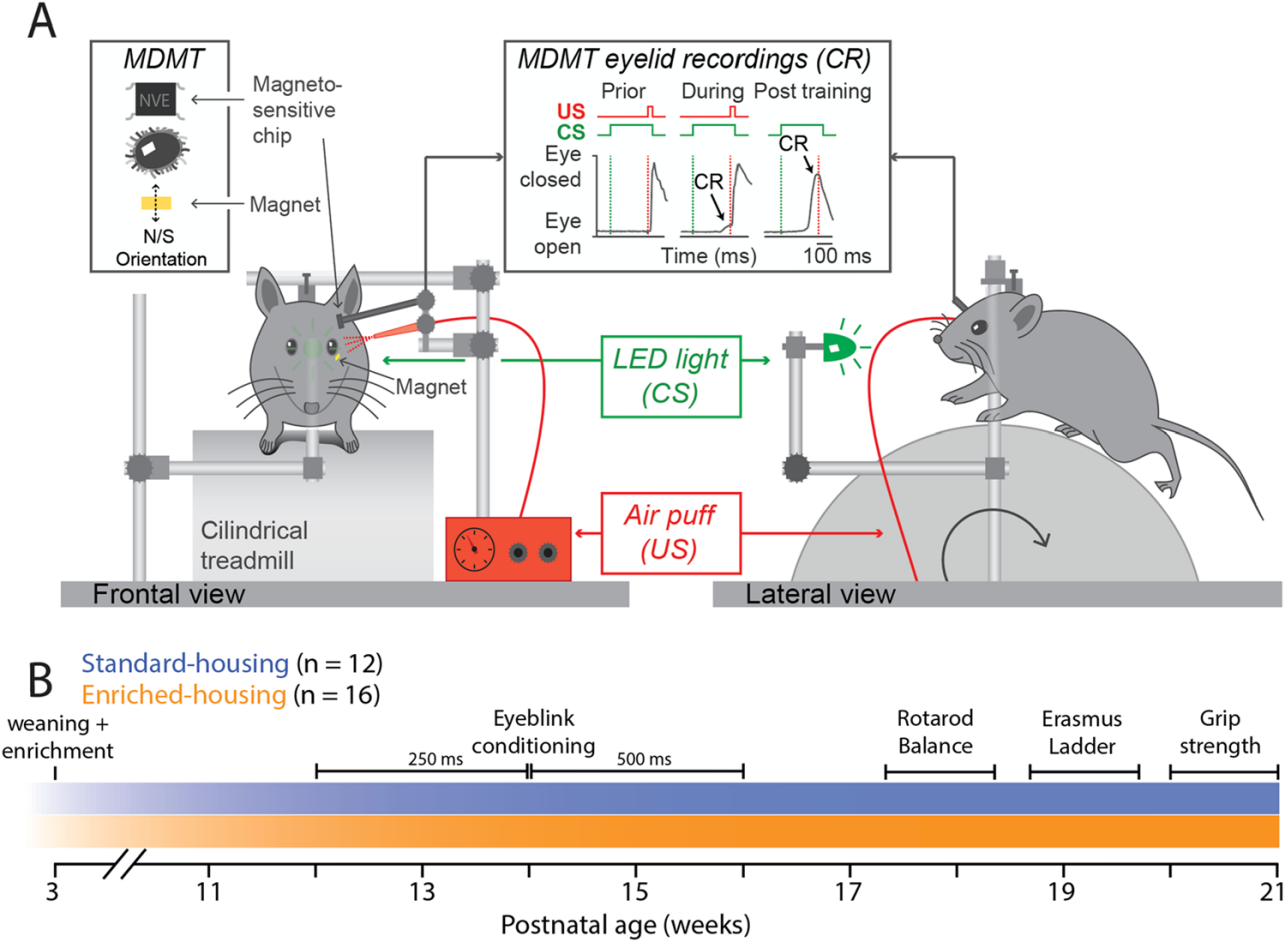
Eyeblink conditioning set-up and experimental timeline. (**A**) Mouse experimental eyeblink conditioning set-up. The conditional stimulus (CS) was a green LED light and the unconditional stimulus (US) was a mild air puff presented to the eye. Eyelid movements were recorded using MDMT, combined with high-speed video recordings (300 fps). During the experiments mice were head-fixed on top of a foam treadmill and able to walk freely. (**B**) Timeline of the performed experiments. From the age of three weeks enriched-housed mice (orange, n = 16) were socially and physically enriched, while standard-housed mice (blue, n = 12) were housed individually only with bedding and nesting material. All mice started with eyeblink conditioning, followed by the accelerating rotarod and balance beam test, then the ErasmusLadder, and finally the grip strength test. Abbreviations: *CR* conditioned response, *CS* conditioned stimulus, *US* unconditioned stimulus.

One suitable paradigm to study the *behavioral effects* of cage enrichment on cerebellar functioning is *Pavlovian eyeblink conditioning*. During this cerebellar learning task, mice first hear a short tone or see a light (conditional stimulus, CS) and several hundred milliseconds later they receive an air-puff on the eye that evokes a reflexive eyeblink (unconditional stimulus, US). As a result of repeated CS–US pairings, mice will eventually learn to close their eyes in response to the CS, which is called the conditioned response (CR). The CR is not simply a static reflex, but instead a precisely timed eyelid movement, the kinetic profile of which is determined by the temporal interval between CS and US (Fig. 1A). Thus, eyeblink conditioning allows the study of both CR probability, as well as the adaptive timing and amplitude of motor responses. Previous studies on the effects of cage enrichment on eyeblink conditioning show mixed results. Rats seem to show faster conditioning^34^, but this effect could not be established in mice^6^. Interestingly, the procedures for eyeblink conditioning in mice have been further improved and optimized over the last decade to make the task less invasive and stressful for the animal. Since prior studies have shown improved spatial learning^35,36^ as well as improved motor coordination by physical factors of enrichment^6,37,38^, one can expect that enrichment may improve various forms of motor learning and performance, including not only acquisition of new sensorimotor associations, but also of general motor abilities that are present from early on. Thus, to achieve a more comprehensive view of cage enrichment effects, we investigated, in addition to eyeblink conditioning, also general motor ability tasks engaging limb movements, including the balance beam and grip strength test, as well as the accelerating rotarod and ErasmusLadder test. Assuming that cage enrichment has positive effects on brain development, improved cognitive function and better overall physical fitness, we hypothesized that cage enrichment in mice leads to both better motor performance and better motor learning.

## Materials and methods

### Subjects, housing, and order of experiments

For all experiments we used male and female C57Bl/6 mice (n = 28, standard-housed mice: 3 females vs. 9 males, enriched-housed mice: 5 females vs. 11 males) housed under a 12-h light/12-h dark cycle, with water and food ad libitum. All experiments were conducted during the light phase, and prior to weaning, all mice were socially housed including the mother, and their cages were equipped with bedding and nesting material. At the age of 3 weeks, litters were randomly divided over two groups: standard-housing and enriched housing. Standard-housed mice (n = 11) were housed individually according to standardized laboratory procedures in a cage that was provided with bedding and nesting material (cage size: 30 × 13 × 13 cm). Enriched-housed mice (n = 16) were socially housed with three to five littermates in a large cage (42 × 26 × 19 cm) and were handled daily by the experimenter for 15 min starting from the age of 3 weeks old. Physical enrichment also started by the age of three weeks and consisted of mouse running wheels, climbing rods, walking bridges, tubes, shelter places, wooden sticks, and nesting materials. To maintain curiosity, these objects were replaced weekly.

All animals were subjected to five different behavioral paradigms in the same order: (1) eyeblink conditioning; (2) balance beam; (3) grip strength test; (4) accelerating rotarod; and (5) ErasmusLadder (Fig. 1B). All methods performed were granted approval in alignment with the European Communities Council Directive for animal experiments, and in accordance with the guidelines and regulations established by the Institutional (Erasmus MC) Animal Care and Use Committee under work protocol: 115-13-08. The study has been conducted in compliance with the ARRIVE guidelines.

### Eyeblink conditioning

The surgical procedure, eyeblink conditioning experiments and applied data analysis used during this study have been described in detail previously^39^.

#### Surgery

In short, to enable head-fixation during eyeblink conditioning experiments, mice underwent pedestal placement surgery. Mice were anesthetized with an isoflurane/oxygen mixture (5% for induction, 1.75–2% for maintenance), while body temperature was kept constant at 37 °C. Eyes were protected against drying using an eye lubricant (Duratears). We made a sagittal scalp incision of 2–3 cm length, then carefully removed the exposed periosteum and roughened the surface of the skull using an etchant gel (Kerr, Bioggio, Switzerland). After this, a small messing block (1.0 × 0.4 × 0.3 mm) with 1 screw thread and 2 additional pinholes was placed on the skull using Optibond eXTRa Universal (Kerr, Bioggio, Switzerland), and dental cement Charisma (Heraeus Kulzer, Armonk, NY, USA). The surgical placement of this so-called pedestal allowed for head fixation during the eyeblink conditioning experiments. Surgeries typically took 15–20 min. After surgery, mice were given 3–5 days to recover.

#### Experimental setup

For eyeblink conditioning, mice were placed head-fixed on top of a cylindrical treadmill on which they were allowed to walk freely^39–41^ (Fig. 1A). Eyelid movements were recorded using MDMT, combined with high-speed video recordings (300 fps) with a Basler camera (Basler ace 750-30gm). Because of the planned transition from MDMT to camera-based data collection, the Basler camera was incorporated into the setup. However, it’s important to note that only the MDMT signal was utilized for the purpose of data analysis (for MDMT details, see Koekkoek et al.,^42^). All stimuli and measurement devices were controlled by National Instruments hardware and custom written LabVIEW software. The CS was a green LED light (LED diameter, 5 mm) placed 10 cm in front of the mouse’s head. Because we performed our experiments in almost complete darkness, this small LED light was a salient stimulus, which could be easily detected by both eyes. The US consisted of a 30 ms duration mild corneal air puff, which was controlled by an API MPPI-3 pressure injector and delivered via a 27.5 mm gauge needle that was perpendicularly positioned at about 10 mm from the center of the left cornea (30–40 psi on pressure injector). We used a random intertrial interval that ranged between 8 and 12 s.

#### Eyeblink conditioning training paradigm

Mice had three days to habituate to the eyeblink setup. During the first two habituation days, the air puff nozzle (for US delivery) and green LED (for CS delivery) were positioned properly but no stimuli were presented^39^. On the third day of habituation, each animal first received 20 CS-only trials and 2 US-only trials as a baseline measure, to establish that the CS did not elicit any reflexive eyelid closure. After the habituation, mice were trained for 20 consecutive daily days. During each training day, mice received in total 240 trials separated over 20 blocks. Each block consisted of 1 CS only, 1 US only, and 10 paired CS–US trials, semi-randomly distributed over the block. A training day lasted for 45–60 min. During the first ten days of training, the duration of the CS was 280 ms and the interval between CS and US onset was set at 250 ms, during the second ten days of training, the duration of the CS was 530 ms and the interval between CS and US onset was 500 ms. Because of an inherent 14-ms delay in the delivery of the air puff, we triggered the air puff at 236 (for the 250 ms ISI during day 1–10) or 484 ms (for the 500 ms ISI during day 11–20) after CS onset so that it would hit the cornea exactly at 250 ms after CS onset. The intertrial interval (ITI) was set according to the following constraints: a random interval between 8 and 12 s had to elapse, the eyelid had to be open below a predetermined threshold of 50% of a full eyelid closure, and eyelid position had to be stable for at least 2 s for a trial to begin. During all training days, the experimenter carefully inspected threshold and stability parameters and adjusted them if necessary. All experiments were performed at approximately the same time of day by the same experimenter.

#### Data analysis

Individual eyeblink traces were analyzed with custom computer software (R Studio; Boston, MA)^39^. For analysis of conditioned behavior, we only included CS-only trials since they show the full kinetic profile of the eyeblink CR providing better information about the adaptive timing of eyeblink CRs. MDMT was utilized to capture all eyelid movement^42^. By applying a voltage to the sensor, the resulting voltage is determined by the strength of the magnetic field. Proper calibration enables the voltage to directly indicate the distance between the magnet and the sensor. The acquired signals undergo scrutiny according to the specified description. Trials with significant activity in the 500 ms pre-CS period (> 7-times the interquartile range) were regarded as invalid and disregarded for further analysis. Trials were normalized by aligning the 500 ms pre-CS baselines and normalizing the signal so that the size of a full blink was 1. This normalization was achieved by using the reflexive blinks to the air-puff (unconditioned responses, UR) as a reference. For each day, we calculated the maximum value in the averaged UR and individual traces were normalized by dividing each trace by this value. As a consequence, in the normalized traces, a value of 1 corresponded with the eye being fully closed, a value of 0 corresponded with the eye being fully open. In valid normalized CS-only trials, all eyelid movements larger than 0.1 and with a latency to CR onset between 50 and 500 ms and a latency to CR peak between 100 and 1000 ms (both relative to CS onset) were considered a CR. We used the same criteria for CR detection for CS-only trials as those presented during day 1–10 with the 250 ms ISI and CS-only trials presented during day 11–20 with the 500 ms ISI. Based on these criteria, we calculated the following outcomes: (1) CR probability for each mouse for each day; (2) the maximum amplitude of the normalized eyelid closure (NEC) in 100–1000 ms interval after CS onset calculated over *all* trials (NEC_all_trials_); (3) the maximum amplitude of the normalized eyelid closure in 100–1000 ms interval after CS onset calculated over trials with a CR (NEC_CR_trials_); (4) the latency to CR onset in trials wherein a CR was present; (5) the latency to CR peak in trials wherein a CR was present; and (6) the percentage of “perfectly-timed CRs”, which were defined as CRs with a latency to the peak of the CR occurring at the onset of the expected US (250 ± 50 ms for the ISI 250 ms, and 500 ± 100 for the ISI 500 ms). Outcomes (4)–(6) were evaluated to quantify the adaptive timing of eyeblink CRs.

Statistical analysis was done using multilevel linear mixed-effects (LME) models in R Studio (code available upon request), we used day and group as a fixed effect, and mouse as a random effect. Goodness of fit model comparison was determined by evaluating log likelihood ratio, BIC, and AIC values. The distribution of residuals was inspected visually by plotting the quantiles of standard normal versus standardized residuals (i.e., Q–Q plots). Data were considered as statistically significant if the (adjusted) *p *value was smaller than 0.05.

### Balance beam

Balance and motor coordination were assessed using the balance beam test^43^. This test consisted of two elevated platforms within between either a 6- or 12 mm diameter round wooden beam (Fig. 4A). Mice had to walk across the beam to a safe end platform—in our case their home cage—and were trained over 3 consecutive days: 2 days of training and 1 day of testing. On the first day of training, mice had to cross the 12 mm beam three times. On the second day of training, mice first had to cross the12 mm beam once more followed by three crossings of the 6 mm beam. The third day of training was the ‘test day’, during which mice first had to cross twice the 12 mm beam, followed by two crossings of the 6 mm beam. Our dependent variable was the time it took for a mouse to cross the beam (two values per mouse for each beam, four values in total).

Statistical significance was determined using linear mixed effect models (LME) in R studio), with beam width, group and their interaction as fixed effects and mouse as a random effect. Data were considered statistically significant if the Bonferroni corrected p-value was smaller than 0.05.

### Grip strength test

The grip strength test was used to determine peak muscle strength of the forelimbs^44^. The test consisted of a grid with horizontal bars attached to a force gauge (BIOSEB, Chaville, France). The grip strength was measured by gently pulling the tail backwards, while the mouse grabbed the bar from the grid with both forelimbs (Fig. 4C). Mice received two daily trials over four consecutive days. Our dependent variable was the maximum grip strength of the mouse just before releasing the grid (8 values in total for each mouse).

Statistical significance was determined using linear mixed effect models (LME) in R studio, with group as a fixed effect and mouse as a random effect. Data were considered statistically significant if the adjusted *p *value was smaller than 0.05.

### Accelerating Rotarod

Motor coordination and motor learning were assessed using the accelerating rotarod test (Ugo Basile, Comerio Varese, Italy)^45^. The rotarod consisted of an accelerating rod with five lanes and a monitoring device (Fig. 4E). Mice had to walk on the rod, while the rod was set to accelerate from 4 to 40 rpm over 270 s; the maximal walking time was set at 300 s. The monitoring device automatically registered the time when the mouse fell off the rotating rod. It was also considered a “fall” when the animal stopped walking, and clung to the rod for three consecutive rotations. Mice were tested over four consecutive days, each day receiving four trials. Our dependent variable was the latency to fall off the rod (4 values for each mouse for 4 days, 16 values in total).

Statistical significance was determined using linear mixed effect models (LME) in R studio, with group and day as fixed effects and mouse and trial as a random effect. Data were considered statistically significant if the adjusted *p *value was smaller than 0.05.

### ErasmusLadder

The ErasmusLadder (Noldus B.V.) is a fully automated device, which detects deficits in motor performance and motor learning^46,47^. The ErasmusLadder consisted of two shelter boxes equipped with: pressurized air outlets and LED lights, and in between a ladder. Mice had to cross the ladder which consisted of 74 rungs, 32 rungs on each side. All rungs are equipped with pressure sensors monitoring the walking pattern. High and low positions of the rungs vary on both sides, across from each high rung is a lower rung. The most convenient walking pattern is the use of the higher rungs, so-called correct steps, the use of lower rungs were considered as missteps (Fig. 5A).

At the beginning of each training the mouse was placed in the “start” box waiting for a LED light to turn on, followed a few milliseconds later, by a gradually becoming more powerful airflow until the mouse entered the “end” box. Once the “end” box was entered, the air blowing stopped and the “inter trial interval” period started. This interval period varied between 8 and 12 s and mice had to stay in that current box. As soon as the mouse escaped the box, air was blown from the opposite shelter box to force the animal to go back. Mice were trained for five consecutive training days, whereby each daily training consisted of 42 runs. Our dependent variable was the percentage of correct steps per run (42 percentage values for each mouse per day, 210 values for each mouse in total).

Statistical significance was determined using linear mixed effect models (LME) in R studio), with group and day as fixed effects and mouse and run as a random effect. Data were considered statistically significant if the adjusted *p* value was smaller than 0.05.

## Results

### Pavlovian eyeblink conditioning

The standard-housed mice (n = 12) and enriched-housed mice (n = 16) were initially trained for ten consecutive days with an interval of 250 ms between the CS and US onsets. Subsequently, both groups were trained for another ten consecutive days with a longer interstimulus interval (ISI) of 500 ms (Fig. 1), which is known to be more challenging for mice^48,49^.

#### Normalized eyelid closure: all trials

**Figure 2:**
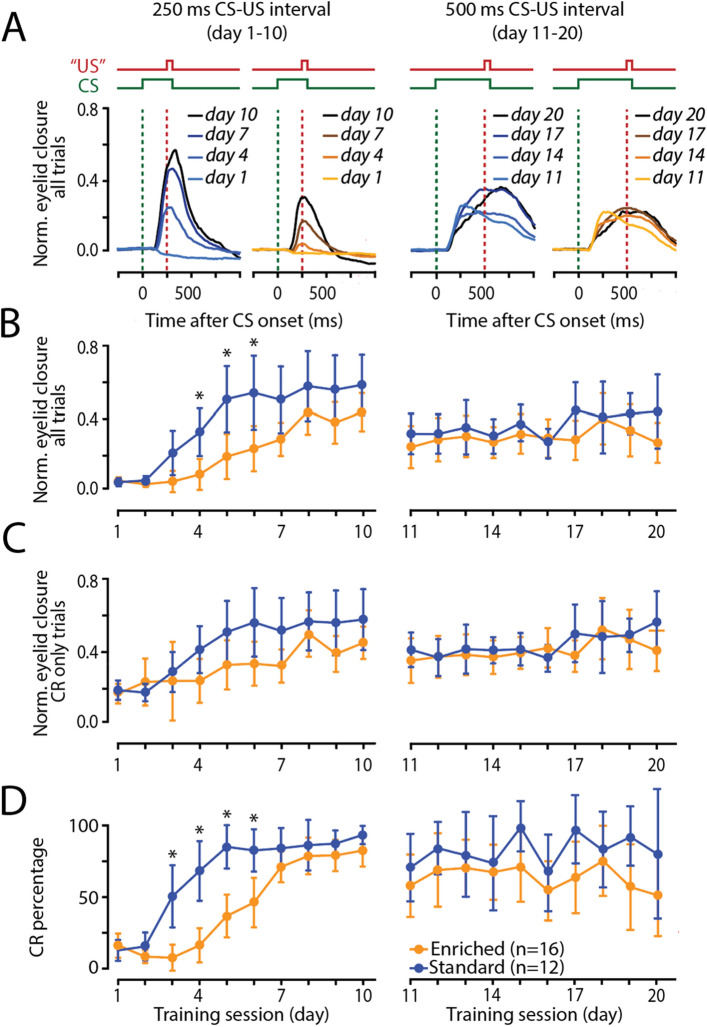
Enriched-housed mice have slower acquisition of eyeblink conditioned responses than standard-housed mice. (**A**) Group averaged eyeblink traces (blue: standard-housed mice, n = 12; orange: enriched-housed mice, n = 16) of four training sessions during the 250 ms ISI paradigm and four training sessions during the 500 ms ISI paradigm. (**B**) Normalized eyelid closure calculated over all trials (NEC_all_trials_) for the first ten days of training with and ISI of 250 ms (left panel), and second ten days of training with an ISI of 500 ms. Standard-housed mice showed faster learning in the 250 ms ISI. (**C**) Normalized eyelid closure calculated over trials with a CR (NEC_CR_trials_) for the first ten days of training with and ISI of 250 ms (left panel), and second ten days of training with an ISI of 500 ms. No group differences were found. (**D**) Percentage of conditioned responses for the first ten days of training with and ISI of 250 ms (left panel) and second ten days of training with an ISI of 500 ms. the 500 ms ISI (day 11–20). Standard-housed mice showed faster learning in the 250 ms ISI. Abbreviations: *CR* conditioned response, *CS* conditioned stimulus, *US* unconditioned stimulus. All error bars represent the 95% confidence interval. *Significance level *p* < 0.05 after correction Bonferroni-Holm.

We evaluated the normalized eyelid closure over all trials (NEC_all_trials_) for the first ten days of training with the ISI of 250 ms (Fig. 2A). In standard-housed mice, the NEC_all_trials_ started at 0.03 (± 0.02) on day 1 and increased to 0.53 (± 0.16) on day 10 (Supplementary Table 1). In the enriched-housed mice, NEC_all_trials_ increased from 0.03 (± 0.02) on day 1 to 0.39 (± 0.10) on day 10. All values represent mean ± 95% confidence interval. We found a significant effect of ‘group’ (F_(1,26)_ = 9.44, *p* = 0.0049), ‘training day’ (F_(9,233)_ = 16.45, *p* < 0.0001), and ‘group * training day’ interaction (F_(9,233)_ = 2.33, *p* = 0.0159). Post hoc testing and correction for multiple comparisons using Holm-Bonferroni, revealed that enriched-housed mice showed statistically significant lower values for NEC—all trials at training day 4 (*p* = 0.0456), day 5 (*p* = 0.0040), day 6 (*p* = 0.0045) (Fig. 2B, Supplementary Table 1). After the switch to the longer interstimulus interval of 500 ms, standard-housed mice had a NEC_all_trials_ of 0.32 (± 0.11) on day 11 and 0.44 (± 0.20) on day 20, whereas enriched-housed mice started with 0.25 (± 0.11) on day 11 and ended with 0.27 (± 0.11) on day 20. We could not establish a significant effect of ‘group’ (F_(1,26)_ = 1.12, *p* = 0.3003), ‘training day’ (F_(9,228)_ = 1.50, *p* = 0.1484) or ‘group * training day’ interaction (F_(9,228)_ = 0.05, *p* = 0.4042) (Fig. 2B, Supplementary Table 2).

#### Normalized eyelid closure: CR trials

Next, we looked into the normalized eyelid closure in CR positive trials (NEC–CR trials) for the first ten days of training with the 250 ms ISI. In standard-housed mice, the NEC_CR_trials_ started at 0.19 (± 0.05) on day 1 and increased to 0.58 (± 0.16) on day 10 (Supplementary Table 3). In the enriched-housed mice, NEC_CR_trials_ increased from 0.17 (± 0.05) on day 1 to 0.45 (± 0.09) on day 10. All values represent mean ± 95% confidence interval. We found a significant effect for ‘training day’ (F_(9,196)_ = 9.62, *p* ≤ 0.0001), and a trend for the effect of ‘group’ (F_(1,26)_ = 3.95, *p* = 0.0576) and ‘group * training day’ interaction (F_(9,196)_ = 1.68, *p* = 0.0953) during the 250 ms ISI paradigm (Fig. 2C, Supplementary Table 3). Post hoc testing and correction for multiple comparisons using Holm-Bonferroni did not show a statistical effect for NEC–CR trials. After the switch to the longer interstimulus interval of 500 ms, standard-housed mice started with NEC_CR_trials_ at 0.41 (± 0.09) on day 11 and 0.56 (± 0.17) on day 20, whereas enriched-housed mice started at 0.35 (± 0.12) on day 11 and ended with 0.41 (± 0.11) on day 20. A significant effect was established for ‘training day’ (F_(9,224)_ = 2.34, *p* = 0.0155), but no effect of ‘group’ (F_(1,26)_ = 0.30, *p* = 0.5896) or ‘group * training day’ interaction (F_(9,224)_ = 1.15, *p* = 0.3255) (Fig. 2C, Supplementary Table 4).

#### CR percentage

Then, we evaluated the percentage of conditioned responses for the first ten days of training with the 250 ms ISI. In standard-housed mice, the CR percentage started at 11.64 (± 7.31) on day 1 and increased to 92.34 (± 6.35) on day 10 (Fig. 2D, Supplementary Table 5). In the enriched-housed mice, CR percentage increased from 15.25 (± 8.76) on day 1 to 81.44 (± 11.20) on day 10. All values represent mean ± 95% confidence interval. We found a significant effect of ‘group’ (F_(1,26)_ = 15.14, *p* = 0.0004), ‘training day’ (F_(9,233)_ = 52.05, *p* < 0.0001) and the ‘group * training day’ interaction (F_(9,233)_ = 7.83, *p* < 0.0001) during the 250 ms ISI paradigm. Post hoc testing and correction for multiple comparisons using Holm–Bonferroni, revealed that enriched-housed mice showed a statistically significant decrease in the CR percentage on training days 3–5 (*p* = 0.0010) and day 6 (*p* = 0.0014) (Supplementary Table 5). After the switch to the longer interstimulus interval of 500 ms, standard-housed mice reached a CR percentage of 71.21 (± 14.20) on day 11 and 76.77 (± 27.01) on day 20, whereas enriched-housed mice started with 63.62 (± 13.16) on day 11 and ended with 59.54 (± 17.21) on day 20 (Fig. 2D, Supplementary Table 6). We could not establish a significant effect of ‘group’ (F_(1,26)_ = 2.06, *p* = 0.1634), ‘training day’ (F_(9,228)_ = 1.80, *p* = 0.0684) nor ‘group* training day’ interaction (F_(9,228)_ = 0.78, *p* = 0.6386) (Fig. 2D, Supplementary Table 6).

#### Adaptive timing

**Figure 3:**
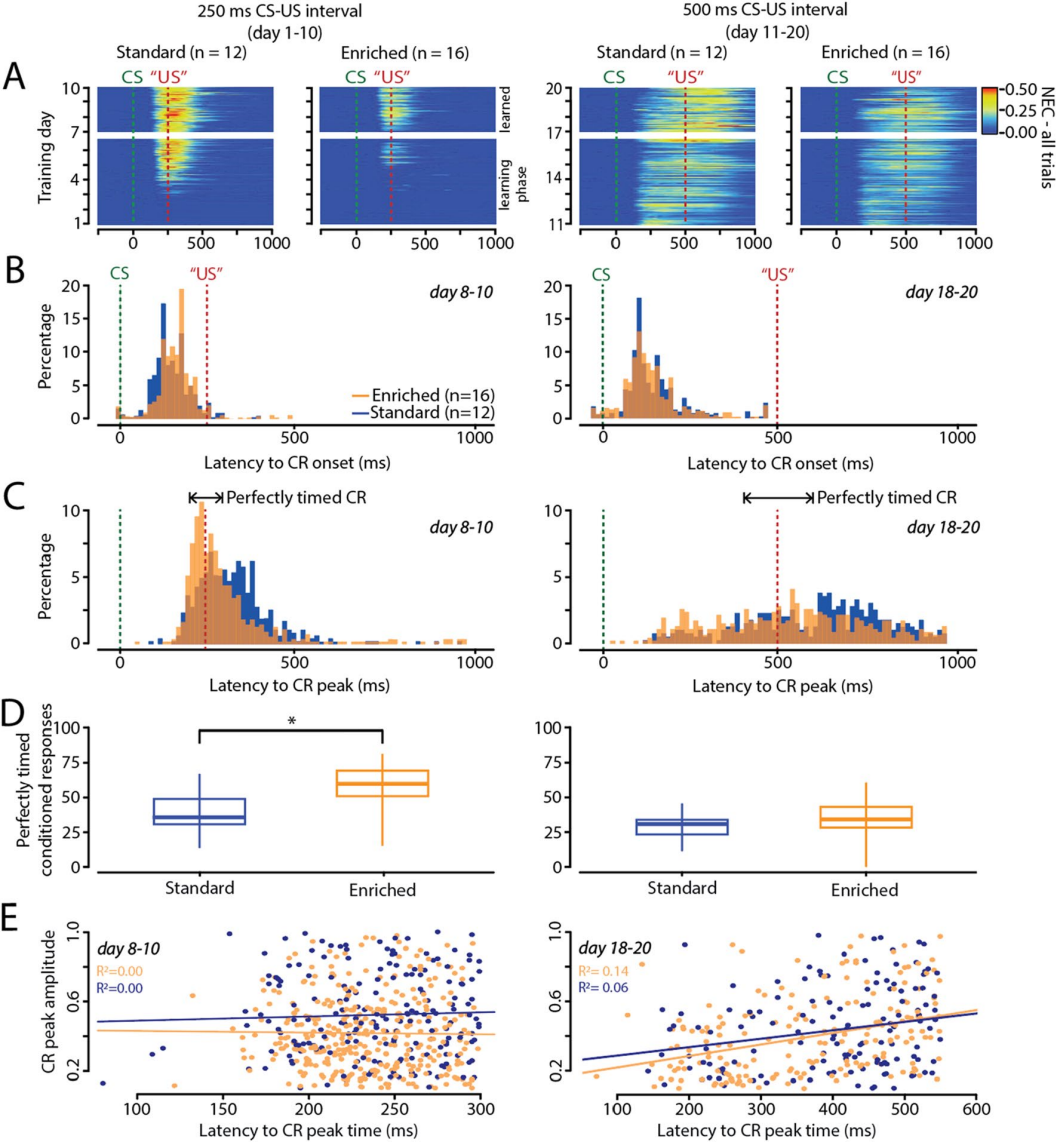
Enriched-housed mice have slightly improved timing of their eyeblink conditioned responses than standard-housed mice. (**A**) Heat map showing the amplitude of conditioned responses (CR) per group. (**B**) Distribution of the latency to CR onset for training days 8–10 for the 250 ms ISI, and day 18–20 for the 500 ms ISI training paradigm. (**C**) Distribution of the latency to CR peak for training days 8–10 for the 250 ms ISI, and day 18–20 for the 500 ms ISI training paradigm. Note that in enriched-housed animals the CRs are more closely centered around the onset of the expected US for the 250 ms ISI paradigm. We defined a “perfectly timed” CR window, indicated with the black arrows. (**D**) Enriched-housed mice show a higher percentage of perfectly timed CR than standard-housed animals. This effect was only observed for the shorter 250 ms ISI training paradigm. (**E**) Correlation between latency to CR peak amplitude and peak timing for days 8–10 for the 250 ms ISI, and day 18–20 for the 500 ms ISI training. The blue line represents the regression line for the standard-housed mice, and the orange line represents the regression line for the enriched-housed mice. Abbreviations: *CR* conditioned response, *CS* conditioned stimulus, *US* unconditioned stimulus. *Significance level *p* < 0.05 after correction Bonferroni-Holm.

Next, we assessed the adaptive timing of eyeblink CRs (Fig. 3A), i.e. the latency to CR onset and latency to CR peak. For the first ten days of training with the 250 ms ISI, standard-housed mice started with a latency to CR onset at 185.38 (± 119.31) on day 1 and ended at 145.92 (± 33.40) on day 10 (Fig. 3B, Supplementary Table 7). The enriched-housed mice started with a latency to CR onset at 259.03 (± 91.96) on day 1 and ended at 161.43 (± 17.17) on day 10. All values represent mean ± 95% confidence interval. The latency after CR onset revealed a significant effect of ‘group’ (F_(1,26)_ = 7.23, *p* = 0.0123) and ‘training day’ (F_(9,196)_ = 2.76, *p* = 0.0047), but not of ‘group * training day’ interaction (F_(9,196)_ = 1.60, *p* = 0.1186) (Fig. 3B). Post hoc testing and correction for multiple comparisons using Holm-Bonferroni, revealed a statistical significance on day 3 (*p* = 0.0030) driven by the standard-housed mice (Supplementary Table 7). Standard-housed mice showed overall consistent adaptive timing of CRs, while enriched-housed mice showed a steeper learning curve. After the switch to the longer 500 ms interstimulus interval, standard-housed mice started with a latency to CR onset at 142.50 (± 21.99) on day 11 and ended at 188.36 (± 25.72) on day 20 (Fig. 3B, Supplementary Table 8). The enriched-housed mice started with a latency to CR onset at 177.11 (± 30.66) on day 11 and ended at 185.36 (± 31.31) on day 20. The latency to CR onset revealed a significant effect of ‘training day’ (F_(9,224)_ = 5.05, *p* < 0.0001), but not for ‘group’ (F_(1,26)_ = 1.08, *p* = 0.3076) or the ‘group * training day’ interaction (F_(9,224)_ = 0.55, *p* = 0.8395) (Supplementary Table 8).

We evaluated the latency to CR peak, which is the latency to maximum eyelid closure relative to CS onset, for the first ten days of training with the 250 ms ISI. Standard-housed mice started with a latency to CR peak at 448.60 (± 235.23) on day 1 and decreased to 325.84 (± 33.41) on day 10 (Fig. 3C, Supplementary Table 9). The enriched-housed mice started with a latency to CR peak at 530.54 (± 140.86) on day 1 and decreased to 303.70 (± 49.79) on day 10. All values represent mean ± 95% confidence interval. We found a significant effect of ‘training day’ (F_(9,196)_ = 3.47, *p* = 0.0005) and ‘group * training day’ interaction (F_(9,196)_ = 2.00, *p* = 0.0418), but not for ‘group’ (F_(1,26)_ = 1.93, *p* = 0.1765) during the 250 ms ISI paradigm. Post hoc testing and correction for multiple comparisons using Holm-Bonferroni, revealed a statistical significance on day 3 (*p* = 0.0030), driven by better timing of enriched-housed mice (Fig. 3C, Supplementary Table 9). After the switch to the longer interstimulus interval of 500 ms, standard-housed mice showed a latency to CR peak started at 396.63 (± 83.67) on day 11 and 644.17 (± 67.70) on day 20, whereas enriched-housed mice started with 377.43 (± 72.92) on day 11 and ended with 544.50 (± 108.76) on day 20. We established a significant effect for latency to CR peak of ‘training day’ (F_(9,224)_ = 10.95, *p* < 0.0001), but not for ‘group’ (F_(1,26)_ = 0.79, *p* = 0.3810) or the ‘group * training day’ interaction (F_(9,224)_ = 0.76, *p* = 0.6514) (Supplementary Table 10). We analyzed the overall latency to CR peak at the end of training (Fig. 3C, Day 8–10 for ISI 250 ms and day 18–20 for ISI 500 ms) with a model including group as a fixed effect and CR amplitude as a covariate. This analysis revealed no effect for 250 ms ISI, but a significant effect of CR amplitude on response time for the 500 ms interval (*p* = 0.0811 for ISI 250 ms and *p* ≤ 0.0001 for ISI 500 ms). The effect of group did not reach statistical significance (*p* = 0.4030 for ISI 250 ms and *p* = 0.1160 for ISI 500 ms), suggesting that the observed variability in response time was predominantly driven by differences in CR amplitude rather than group. These findings highlight the primary role of CR amplitude in explaining the observed variability in CR response time, with group differences having a relatively minor impact.

We also analyzed the “perfectly timed” CR’s defined as CRs that peak in the very close proximity of the expected onset of the US (Day 8–10: 250 ± 50 ms; Day 18–20: 500 ± 100 ms). We found that during the 250 ms ISI paradigm, standard-housed mice showed a percentage of 39.39 (± 4.47) perfectly timed CRs and enriched-housed mice of 57.56 (± 4.17). All values represent mean ± 95% confidence interval. The percentage of perfectly timed CRs showed a significant effect for ‘group’ (F_(1,26)_ = 5.30, *p* = 0.0296), driven by better timing of enriched-housed mice (Fig. 3D, Supplementary Table 11). After the switch to the longer interstimulus interval of 500 ms, standard-housed mice showed a percentage of 28.69 (± 4.66) perfectly timed CRs, and enriched-housed mice of 35.92 (± 4.65). For this longer interval no significant effect was found on the perfectly timed CRs for ‘group’ (F_(1,26)_ = 1.31, *p* = 0.2630) (Supplementary Table 12).

Next, we analyzed the correlation between CR peak amplitude and peak timing precision to investigate whether CR timing precision is influenced by variations in CR amplitude or remains independent (Fig. 3E). Additionally, we examined the timing precision of CRs specifically in standard housing mice, focusing on CRs that closely matched the amplitudes observed in enriched housing mice. To achieve this, we utilized the interquartile range of CR amplitudes from enriched animals as a benchmark, which fell between 0.3 and 0.6 for an ISI of 250 ms and between 0.2 and 0.6 for an ISI of 500 ms. Similar to our analysis of "perfectly timed CRs" without this criterion, we observed a statistically significant group effect for an ISI of 250 ms (F_(1,26)_ = 7.21, *p* = 0.0124), but no significant effect for an ISI of 500 ms (F_(1,25)_ = 0.30, *p* = 0.5873). Consequently, we conclude that timing precision at the 250 ms ISI in our experiment is primarily influenced by group differences, with the contribution of CR amplitude being less pronounced.

Based on these combined results, we conclude that environmental enrichment in mice does not enhance learning speed in eyeblink conditioning, but slightly improves the adaptive timing of eyeblink CRs.

### Balance beam test

**Figure 4:**
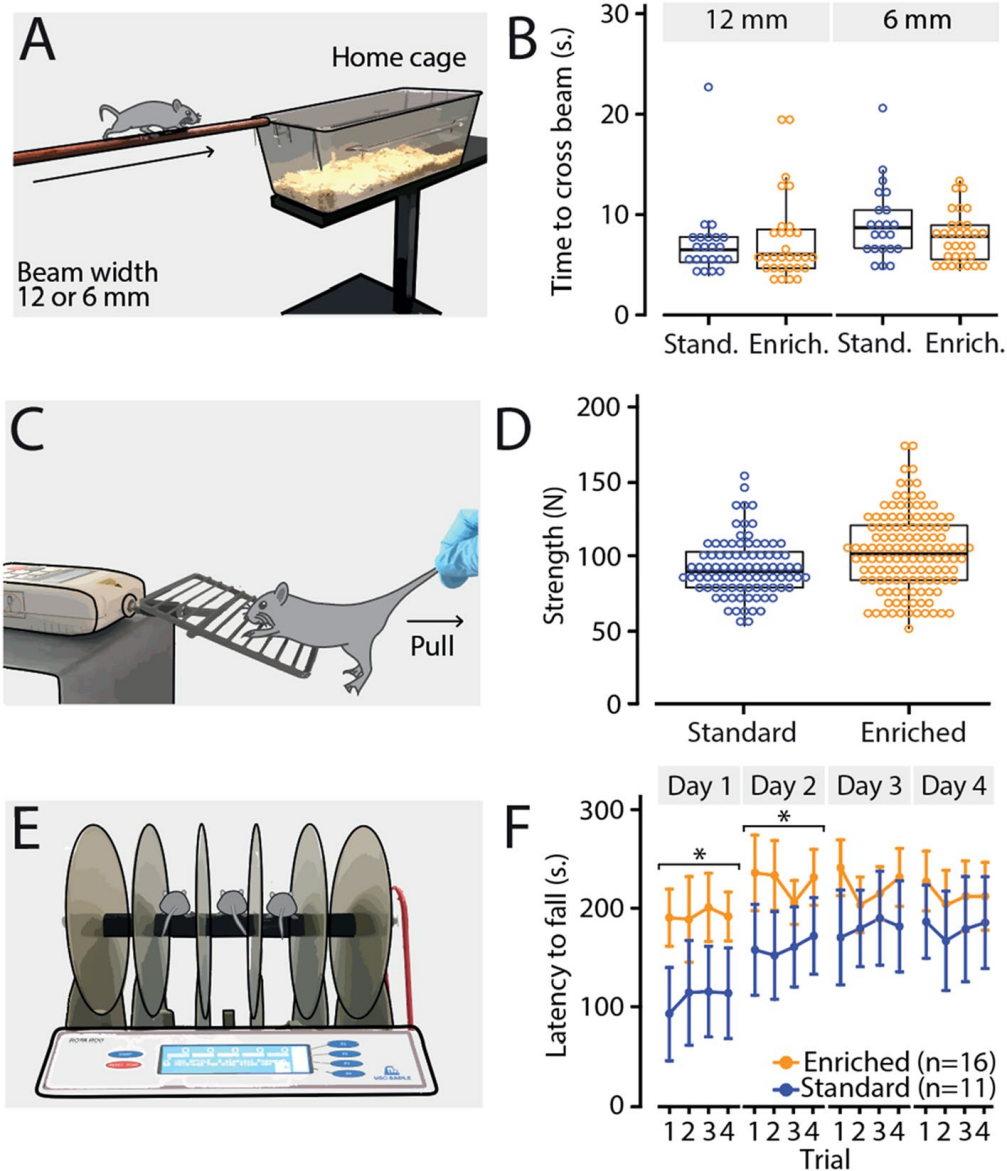
Enriched-housed animals perform better on accelerating rotarod, but not on the balance beam or grip strength tests. (**A**) Illustration of the balance beam test, where time crossing the beam from one side to the other was quantified on two different beam widths: 6 mm and 12 mm. (**B**) Boxplots of the average time (s) on the beam per group and beam width. Each dot represents a single value per mouse, with in total two values per beam width per mouse. (**C**) Illustration of the grip strength test, where the maximal muscle strength of the forelimbs was quantified by grabbing a bar from the grid before releasing. (**D**) Boxplots of the average peak strength (N) per group. Each dot represents a single value per mouse, with 8 values in total for each mouse. (**E**) Illustration of the accelerating rotarod, where the latency to fall was quantified. Mice had to walk on the accelerating rod (4–40 RPM) for a maximum of 300 s. (**F**) Line plot represent the average latency to fall (s) per trial per day, and error bars representing a 95% confidence interval. The blue data represents standard-housed mice, where the orange data represents the enriched-housed mice. *Significance level *p* < 0.05 after correction Bonferroni-Holm.

To test motor balance, standard-housed control mice (n = 11) and enriched-housed mice (n = 16) performed the balance beam test (Fig. 4A). We analyzed the time it took for each mouse to cross the beam, for both a 6- and 12-mm width beam. To determine statistical significance, we used a linear mixed-effect (LME) model with ‘group’, ‘beam width’, and ‘group * beam width’ interaction used as fixed effects and ‘mouse’ as a random effect. For the 12 mm wide beam, we found that standard-housed mice needed on average 6.48 (± 1.67) seconds and enriched mice needed on average 6.03 (± 1.57) seconds to cross the beam (all values: median ± 95% CI). For the narrower 6 mm beam, it took standard-housed control mice on average 8.70 (± 1.65) seconds and enriched mice on average 7.83 (± 1.58) to walk from one side to the other (Fig. 4B). With an ANOVA on our LME, we found a significant effect for ‘beam width’ (F_(1,77)_ = 4.54, *p* = 0.0362), but no effects for ‘group’ (F_(1,25)_ = 0.03, *p* = 0.8654) nor the ‘group*beam width’ interaction (F_(1,77)_ = 0.82, *p* = 0.3671) (Supplementary Table 13). We thus conclude that cage enrichment did not lead to major improvements in motor balance.

### Grip strength test

Muscle strength can have a major effect on motor functioning. We therefore assessed muscle strength of the forelimbs using the grip strength test in standard-housed mice (n = 11) and enriched-housed mice (n = 16) (Fig. 4C). We determined the maximal muscle strength (N) for each mouse. To determine statistical significance, we used a linear mixed-effect model with ‘group’ as a fixed effect and ‘mouse’ as a random effect. We found that the peak muscle strength value of standard-housed mice was on average 90 (± 4.11) Newtons and that enriched mice could deliver on average 102 (± 4.62) Newtons (all values: median ± 95% CI) (Fig. 4D). Although the enriched mice tended to be slightly stronger, the effect of ‘group’ was not statistically significant (F_(1,25)_ = 2.48, *p* = 0.1281) (Supplementary Table 13). We conclude that enriched housing had no major impact on peak muscle strength of the mouse’s forelimbs.

### Accelerating Rotarod

To test motor performance, standard-housed control mice (n = 11) and enriched-housed mice (n = 16) were trained on the accelerating rotarod (Fig. 4E). We analyzed the latency to fall (s) from the rotating rod for each day for 4 consecutive training days. We used a linear mixed effect model with ‘training day’, ‘group’ and ‘training day * group’ interaction as fixed effects and ‘mouse’ as a random effect.

Standard-housed mice were able to walk on the accelerating rod on day 1 for 108.18 (± 48.08) seconds and for 178.68 (± 47.04) seconds on day 4 (for both values: mean ± 95% CI) (Fig. 4F). We found a significant effect of ‘day’ within the standard-housed group (ANOVA on LME, main effect: F_(3,162)_ = 27.61, *p* < 0.0001; post hoc, day 1–2, *p* < 0.0001; day 2–3, *p* = 0.09; day 3–4, *p* = 0.917; correction for multiple comparison using Bonferroni-Holm method) (Fig. 4F; Supplementary Table 13). In contrast, enriched-housed mice started with values of 192.25 (± 33.14) seconds on day 1 and ended with 213.13 (± 34.21) seconds on day 4. Herewith, the enriched mice performed better on day 1 than the standard-housed mice did on day 4. For the enriched mice, we also found a significant effect of ‘day’ (ANOVA on LME, F_(3,237)_ = 6.785, *p* = 0.0002; post hoc, day 1–2, *p* = 0.0003; day 2–3, *p* = 0.63; day 3–4, *p* = 0.54; correction for multiple comparison using Holm-Bonferroni method) (Fig. 4F; Supplementary Table 13). When comparing the two groups, we found a significant effect for ‘training day’ (F_(3,399)_ = 25.28, *p* < 0.0001), ‘group’ (F_(1,25)_ = 9.72, *p* = 0.0046), and the ‘training day * group’ interaction (F_(3,99)_ = 6.65, *p* = 0.0002), whereby enriched mice performed much better than the standard-housed mice. We thus conclude that cage enrichment led to significant improvements in motor performance in mice.

### ErasmusLadder

**Figure 5:**
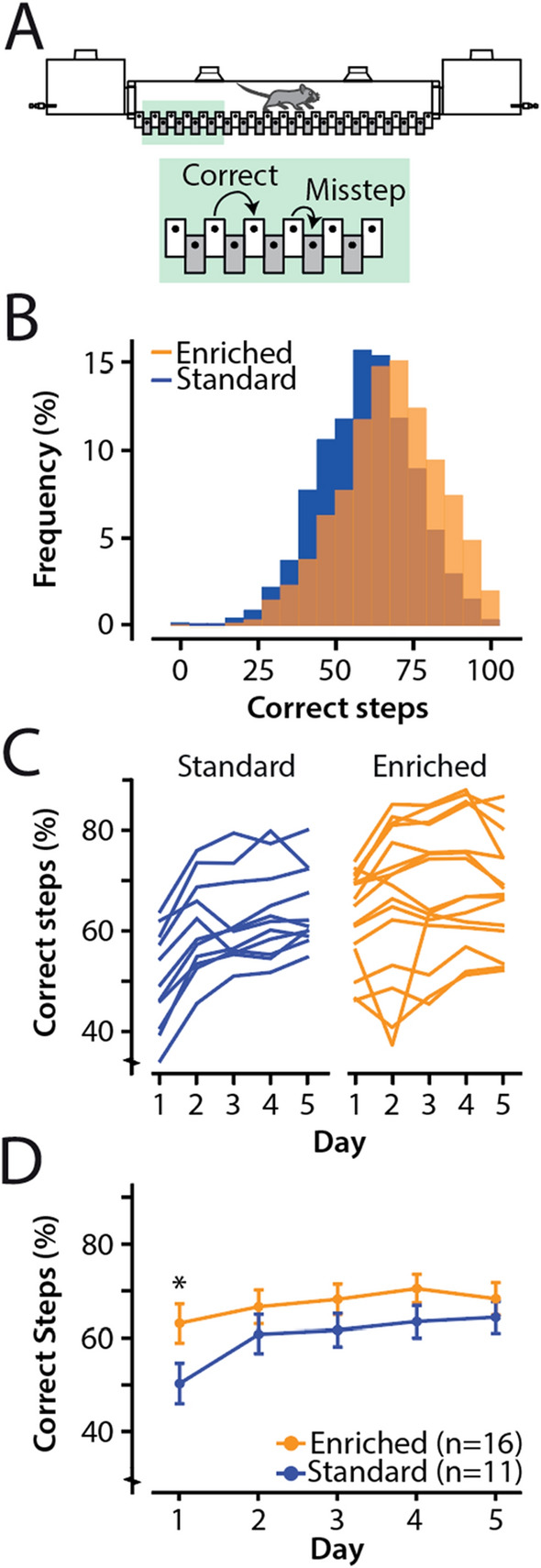
Enriched-housed animals perform better on the ErasmusLadder. (**A**) Illustration of the ErasmusLadder, where mice had to cross the horizontal ladder from one shelter box to the other. The percentage of “correct” steps within each trial was quantified, whereby a correct step was defined as a step of the front-paws from a high rung to the next high rung, irrespective of the length of the step. A step where a lower rung was touched upon, was considered as a misstep. (**B**) Histogram showing the distribution of correct steps for both groups. (**C**) Line plot representing the average correct steps (%) of individual mice (each line represents one animal). (**D**) Line plot representing the average correct steps (%) per group, and error bars representing a 95% confidence interval. The blue data represents standard-housed mice, where the orange data represents the enriched-housed mice. *Significance level *p* < 0.05 after Bonferroni-Holm correction.

To further assess locomotion patterns, standard-housed mice (n = 11) and enriched-housed mice (n = 16) were trained for five consecutive days on the ErasmusLadder. Each training day, mice received 42 trials. Each trial was a crossing of the ladder from one shelter box to the other. We quantified the percentage of “correct steps” within each trial, whereby a correct step was defined as a step of the front-paws from a high rung to the next high rung, irrespectively of the length of the step. A step where a lower rung was touched upon, was considered as a “misstep” (Fig. 5A). Inspection of the correct steps for all training days, revealed a relatively normal distribution for both groups (Fig. 5B, C). We used a linear mixed effect model with ‘group’, ‘day’ and the ‘group*day’ interaction as fixed effects and ‘mouse’ as a random effect.

Standard-housed mice had on average 50.27(± 4.31) percent correct steps on day 1 and 64.32 (± 3.34) percent correct steps on day 5 (all values: mean ± 95% CI) (Fig. 5C). Enriched-housed mice started with an average percentage of correct steps of 63.03 (± 4.14) on day 1 and ended with an average percentage of correct steps of 68.26 (± 3.46) on day 5 (Fig. 5C; Supplementary Table 16). Herewith, the enriched mice performed on day 1 almost as well as the standard-house mice on day 5. We found a significant main effect of ‘day’ (F_(4,5376)_ = 110.99, *p* ≤ 0.0001) and the ‘group * day’ interaction (F_(4,5376)_ = 17.92, *p* ≤ 0.0001), but not for ‘group’ (F_(1,25)_ = 3.26, *p* = 0.0833). Post hoc testing revealed a significant difference between groups on day 1 (*p* = 0.0268), but not for the other days (after correction for multiple comparisons using Holm-Bonferroni) (Fig. 5D; Supplementary Table 16). Together, we conclude that cage enrichment significantly improved locomotion in mice on the ErasmusLadder test.

## Discussion

Motivated to further our understanding of the impact of environmental conditions on our brain activity as well as to improve animal welfare in our laboratory, we investigated the effects of social and physical enrichment of the habitat of C57Bl/6 mice on cerebellar motor learning and performance. We subjected these mice to classical conditioning of eyeblink responses as well as to cerebellar tests that engage limb movements at various levels of complexity, ranging from the balance beam test and grip strength test up to the accelerating rotarod and ErasmusLadder task. During eyeblink conditioning the enriched-housed mice showed a relatively slow acquisition of conditioned eyelid responses (CRs), but during the initial ten days of training with an ISI of 250 ms these mice exhibited a slightly more precise timing in their CRs. Furthermore, these mice showed improved motor performances on the accelerating rotarod as well as the ErasmusLadder, which are some of the more demanding motor coordination tests. In contrast, tasks that are part of daily life from early on, such as walking on a beam or exerting a strong grip, were not affected. Together, these results suggest that environmental enrichment leads to improvements in cerebellar motor learning when the conditions are challenging and require precise timing.

A previous study on associative learning during eyeblink conditioning in mice did not reveal any beneficial effect of either physical or social enrichment^6^. This outcome could be linked to the choice of trace eyeblink conditioning instead of delay conditioning. Moreover, the study exclusively presented results based on the percentage of conditioned responses (CR). Instead, in a similar study in rats, physical enrichment seems to increase not only the learning speed of occurrence of CRs, but also the amplitude of reflexive URs^34^. This might indicate that rats are more responsive to the tone—used as a conditioned stimulus—which may enhance associative fear responses^34^. Likewise, in our hands, the impact of cage enrichment on enriched-housed mice did not exclusively result in positive effects during eyeblink conditioning. Analysis of NEC_all trials_ revealed slower conditioning, consistent with the outcomes of CR percentage where standard-housed mice also showed a better performance almost from the very beginning.

When examining adaptive timing we focused on the latency to CR onset and the timing of the peak amplitude of the CR. Regarding the latency to CR onset, we observed that enriched-housed mice appear to close their eyes relatively late. Even though the latency to CR onset was not significantly longer in enriched housed mice as compared to those raised in the standard housed cages, there appears to be a trend in that direction, raising the possibility that short latency responses (SLRs) linked to stress appear more often without enriched housing^50^. In general, the latency to CR onset in mice appears to be approximately 100 ms after the onset of the conditioned stimulus (CS) and to be unrelated to the CS–US interval. Thereby, our data align well with other studies in mice and rats in which the latency to CR onset remained also relatively constant and was also not influenced by the CS–US interval^48,51^. In this respect, rodents might diverge from other mammals like rabbits where the latency to CR onset appears to be determined at least in part by the CS–US interval^52^.

Regarding the latency to CR peak, which is from a teleological point of view the most relevant timing parameter to measure protection against the intrusive US^53^, we found that enriched-housed mice show signs of better timing during training with an ISI of both 250 ms and that of 500 ms. This was also reflected in the percentage of trials with CRs that were perfectly timed within a narrow time window around the moment when the US is supposed to occur. During the 250 ms ISI paradigm, standard-housed mice showed a significantly lower percentage of perfectly timed CRs compared to the enriched-housed mice. When studying the correlation between CR peak amplitude and the latency to CR peak time, we also observed some signs of significance, allowing us to conclude that environmental enrichment in mice slightly improves their adaptive timing of eyeblink CRs, whereas it does not enhance the acquisition speed of the learning process.

These data raise the question as to why environmental enrichment only leads to improvement in the timing of the eyeblink conditioning in mice? We have several possible explanations. First, it is known that environmental enrichment reduces anxiety and stress-responsive hormones in rodents, such as corticosterone^37,38,54^. Anxiety has large effects on many learning paradigms such as eyeblink conditioning^50,55–58^. Higher anxiety levels affect the ability to perform better under stressed conditions, such as showing a higher rate of conditioning. Second, we observed that enriched-housed mice were more indifferent to the stimuli they received during the eyeblink conditioning experiment. It is known that a US that is perceived as less invasive, leads to slower acquisition of defensive conditioned responses^53^. Indeed, our mice housed in an enriched environment showed slower learning, which could be attributed to their comparatively lower anxiety levels resulting from the environmental enrichment. Third, the advantageous effects of enriched housing appear to affect particularly processes controlled by the spinocerebellum, whereas those that are predominantly regulated by the cerebellar hemispheres, such as eyeblink conditioning, may benefit less^59–62^.

In addition to Pavlovian eyeblink conditioning, we performed tasks that assessed general motor performance using the balance beam test, grip strength test, accelerating rotarod and the ErasmusLadder. In line with prior research, our findings indicated enhanced motor performance patterns in enriched-housed mice, particularly evident in the accelerating rotarod and the ErasmusLadder tasks^6,38^. The enriched-housed mice appear to consistently outperform the standard-housed animals, from the very first day on during both tasks. These enhanced motor skills may be linked to the cage enrichment provided during the weeks prior to the experiments. Even so, the standard-housed animals show a steeper learning curve across sessions, indicating a greater degree of adaptation. Thus, even though enriched-housed mice show improved motor performances from the very start and higher overall levels of performance, it is the standard-housed group that shows better adaptation across sessions.

There was a slight improved trend observed in both the balance beam and grip strength test, but the disparities observed in the enriched-housed mice did not reach statistical significance. These tasks, which are not particularly demanding and basically test behaviors that are in an overtrained state from early on, are probably not sensitive enough to pick up subtle differences between the groups. Similar results were obtained in a study employing larger sample sizes in that a trend in group effects was noticeable, yet no statistically significant effect was found^37^.

Environmental enrichment typically involves increasing motor performance often achieved through the provision of running wheels. Several studies suggest that increased physical activity plays a crucial role in the enrichment’s effects, such as reducing stress. It has been demonstrated that using a wheel alone, without any additional enriching components, is sufficiently adequate to improve learning^22,23,63^. Our results indicate that environmental enrichment, including the use of the running wheel, indeed affected motor learning and improved motor performance on the accelerating rotarod and ErasmusLadder. Yet, during cerebellar associative learning, i.e., Pavlovian eyeblink conditioning, where robust motor performance is not a prerequisite, only adaptive timing showed improvement. One of the explanations might be that mice subjected to the eyeblink paradigm are head-fixed in a dark and soundproof set-up, where they are allowed to walk freely on a wheel. In contrast, during the accelerating rotarod and the ErasmusLadder the animals are in a noisier environment and notably forced to perform. Prior research has indicated that forced running exercise in rodents can induce anxiety and elevate levels of the stress hormone corticosterone in the serum^64–66^. Based on these findings, one could hypothesize that the positive effects of environmental enrichment are primarily evident in forms of learning that necessitate robust motor abilities and generate an optimal level of acute stress.

Limitations of this study include the following: In our experimental design we did not distinguish between physical and social enrichment. Our idea was to notably improve animal well-being by enriching their living conditions in both domains. In addition, we attentionally refrained from measuring of stress hormones in our animals because of the invasive nature of this procedure. In our experimental design, we have made conscious decisions with the aim of investigating the impact on animal behavior through enhancements in animal welfare. Enrichment was provided after weaning, and we specifically choose to conduct all behavioral experiments consistently in the same order to minimize the variables and the number of animals required. The different housing conditions and the order of experiments were applied uniformly to all animals. Furthermore, we have focused on the general impact of environmental enrichment on cerebellar learning as well as the overall animal welfare aspect. For future experiments it will be beneficial to explore potential gender related effects and investigate in a more direct fashion whether enrichment may have differing impacts on stress levels.

In conclusion, our findings indicate that enriched-housed mice show slightly better timing of their conditioned eyeblink responses and outperform animals that are housed in standard conditions in the accelerating rotarod and ErasmusLadder test. Future studies should expand on these basic findings, and further explore the impact of sexes and stress levels.

### Supplementary Information

Supplementary Information.

## Data Availability

The supplementary materials for this study comprise the following datasets: Supplementary Tables 1–12 (eyeblink conditioning data); Supplementary Table 13 (Balance Beam, Grip Strength test and accelerating rotarod); Supplementary Tables 14–16 (ErasmusLadder). The datasets generated and/or analyzed during the current study are available in the Github repository, https://github.com/StephanieDijkhuizen/Data_Environmental_Enrichment.
