# Supplementary material for: Impact of enriched environment on motor performance and learning in mice
Source: Sci Rep. 2024 Mar 12;14:5962. doi: 10.1038/s41598-024-56568-3 (PMC10933351; doi:10.1038/s41598-024-56568-3)
Supplement: Supplementary file 1 — Supplementary Information. [file 41598_2024_56568_MOESM1_ESM.docx]

|  | **Normalized eyelid closure – all trials** | | **Comparisons** | | |
| --- | --- | --- | --- | --- | --- |
| **Day** | **Standard (n=12)** | **Enriched (n=16)** | **p-value** | **Bonf-Holm p-value** | |
| 1 | 0.03 (± 0.02) | 0.03 (± 0.02) | 0.9042 | 1.0000 |  |
| 2 | 0.04 (± 0.02) | 0.02 (± 0.01) | 0.7972 | 1.0000 |  |
| 3 | 0.18 (± 0.12) | 0.03 (± 0.06) | 0.0532 | 0.2660 |  |
| 4 | 0.29 (± 0.12) | 0.07 (± 0.08) | 0.0057* | 0.0456* |  |
| 5 | 0.46 (± 0.17) | 0.16 (± 0.12) | 0.0004* | 0.0040* |  |
| 6 | 0.49 (± 0.19) | 0.20 (± 0.12) | 0.0005* | 0.0045* |  |
| 7 | 0.46 (± 0.17) | 0.25 (± 0.09) | 0.0092* | 0.0644 |  |
| 8 | 0.53 (± 0.18) | 0.40 (± 0.12) | 0.0885 | 0.3540 |  |
| 9 | 0.51 (± 0.18) | 0.34 (± 0.11) | 0.0343 | 0.2058 |  |
| 10 | 0.53 (± 0.16) | 0.39 (± 0.10) | 0.1010 | 0.3540 |  |

| **ANOVA ON LME** |  |  |  |  |
| --- | --- | --- | --- | --- |
| Group | F(1,26) = 9.44, p = 0.0049 |  |  |  |
| Session | F(9,233) =16.45, p = <.0001 |  |  |  |
| Group*Session | F(9,233) = 2.33, p = 0.0159 |  |  |  |

**Supplementary Table 1.** Normalized eye closure calculated over all trials for the 250 ms ISI training paradigm. All values represent mean ± 95% confidence interval.

|  | **Normalized eyelid closure – all trials** | | **Comparisons** | | |
| --- | --- | --- | --- | --- | --- |
| **Day** | **Standard (n=12)** | **Enriched (n=16)** | **p-value** | **Bonf-Holm p-value** | |
| 11 | 0.32 (± 0.11) | 0.25 (± 0.11) | 0.5048 | 1.0000 |  |
| 12 | 0.32 (± 0.11) | 0.29 (± 0.12) | 0.7087 | 1.0000 |  |
| 13 | 0.36 (± 0.15) | 0.31 (± 0.11) | 0.5439 | 1.0000 |  |
| 14 | 0.31 (± 0.09) | 0.27 (± 0.08) | 0.6799 | 1.0000 |  |
| 15 | 0.38 (± 0.10) | 0.32 (± 0.11) | 0.4459 | 1.0000 |  |
| 16 | 0.27 (± 0.08) | 0.30 (± 0.10) | 0.6911 | 1.0000 |  |
| 17 | 0.45 (± 0.15) | 0.29 (± 0.11) | 0.0528 | 0.5280 |  |
| 18 | 0.41 (± 0.20) | 0.40 (± 0.14) | 0.9702 | 1.0000 |  |
| 19 | 0.43 (± 0.11) | 0.34 (± 0.14) | 0.3153 | 1.0000 |  |
| 20 | 0.44 (± 0.20) | 0.27 (± 0.11) | 0.0660 | 0.5940 |  |

| **ANOVA ON LME** |  |  |  |  |
| --- | --- | --- | --- | --- |
| Group | F(1,26) = 1.12, p = 0.3003 |  |  |  |
| Session | F(9,228) = 1.50, p = 0.1484 |  |  |  |
| Group*Session | F(9,228) = 0.05, p = 0.4042 |  |  |  |

**Supplementary Table 2.** Normalized eye closure calculated over all trials at 500 ms after CS onset for the 500 ms ISI training paradigm. All values represent mean ± 95% confidence interval.

|  | **Normalized eyelid closure – CR only** | | **Comparisons** | | |
| --- | --- | --- | --- | --- | --- |
| **Day** | **Standard (n=12)** | **Enriched (n=16)** | **p-value** | **Bonf-Holm p-value** | |
| 1 | 0.19 (± 0.05) | 0.17 (± 0.05) | 0.8085 | 1.0000 |  |
| 2 | 0.18 (± 0.05) | 0.24 (± 0.13) | 0.3354 | 1.0000 |  |
| 3 | 0.29 (± 0.11) | 0.24 (± 0.22) | 0.5479 | 1.0000 |  |
| 4 | 0.41 (± 0.13) | 0.24 (± 0.12) | 0.1763 | 0.9030 |  |
| 5 | 0.51 (± 0.17) | 0.33 (± 0.13) | 0.0339 | 0.2712 |  |
| 6 | 0.56 (± 0.18) | 0.34 (± 0.12) | 0.0056 | 0.0560 |  |
| 7 | 0.51 (± 0.18) | 0.32 (± 0.09) | 0.0238 | 0.2142 |  |
| 8 | 0.57 (± 0.16) | 0.50 (± 0.12) | 0.4113 | 1.0000 |  |
| 9 | 0.56 (± 0.18) | 0.39 (± 0.10) | 0.0459 | 0.3213 |  |
| 10 | 0.58 (± 0.16) | 0.45 (± 0.09) | 0.1505 | 0.9030 |  |

| **ANOVA ON LME** |  |  |  |  |
| --- | --- | --- | --- | --- |
| Group | F(1,26) = 3.95, p = 0.0576 |  |  |  |
| Session | F(9,196) = 9.62, p = <.0001 |  |  |  |
| Group*Session | F(9,196) = 1.68, p = 0.0953 |  |  |  |

**Supplementary Table 3.** Normalized eye closure calculated over the CR only trials for the 250 ms ISI training paradigm. All values represent mean ± 95% confidence interval.

|  | **Normalized eyelid closure – CR only** | | **Comparisons** | | |
| --- | --- | --- | --- | --- | --- |
| **Day** | **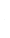Standard (n=12)** | **Enriched (n=16)** | **p-value** | **Bonf-Holm p-value** | |
| 11 | 0.41 (± 0.09) | 0.35 (± 0.12) | 0.5536 | 1.0000 |  |
| 12 | 0.37 (± 0.10) | 0.37 (± 0.11) | 0.9577 | 1.0000 |  |
| 13 | 0.41 (± 0.13) | 0.38 (± 0.11) | 0.6942 | 1.0000 |  |
| 14 | 0.41 (± 0.07) | 0.37 (± 0.09) | 0.6255 | 1.0000 |  |
| 15 | 0.41 (± 0.09) | 0.39 (± 0.09) | 0.7915 | 1.0000 |  |
| 16 | 0.36 (± 0.07) | 0.42 (± 0.11) | 0.4930 | 1.0000 |  |
| 17 | 0.50 (± 0.16) | 0.38 (± 0.09) | 0.1255 | 1.0000 |  |
| 18 | 0.48 (± 0.20) | 0.53 (± 0.17) | 0.5876 | 1.0000 |  |
| 19 | 0.49 (± 0.09) | 0.47 (± 0.16) | 0.6988 | 1.0000 |  |
| 20 | 0.56 (± 0.17) | 0.41 (± 0.11) | 0.0669 | 0.6690 |  |

| **ANOVA ON LME** |  |  |  |  |
| --- | --- | --- | --- | --- |
| Group | F(1,26) = 0.30, p = 0.5896 |  |  |  |
| Session | F(9,224) = 2.34, p = 0.0155 |  |  |  |
| Group*Session | F(9,224) = 1.15, p = 0.3255 |  |  |  |

**Supplementary Table 4.** Normalized eye closure calculated over the CR only trials for the 500 ms ISI training paradigm. All values represent mean ± 95% confidence interval.

|  | **CR percentage** | | **Comparisons** | | |
| --- | --- | --- | --- | --- | --- |
| **Day** | **Standard (n=12)** | **Enriched (n=16)** | **p-value** | **Bonf-Holm p-value** | |
| 1 | 11.64 (± 7.31) | 15.25 (± 8.76) | 0.6848 | 1.0000 |  |
| 2 | 14.41 (± 9.75) | 7.22 (± 4.52) | 0.4037 | 1.0000 |  |
| 3 | 49.42 (± 21.75) | 6.51 (± 9.07) | <.0001 | 0.0010 |  |
| 4 | 67.14 (± 20.83) | 15.17 (± 11.96) | <.0001 | 0.0010 |  |
| 5 | 84.05 (± 15.16) | 35.69 (± 14.94) | <.0001 | 0.0010 |  |
| 6 | 81.54 (± 14.73) | 45.07 (± 17.29) | <.0002 | 0.0014 |  |
| 7 | 83.00 (± 14.16) | 70.02 (± 10.74) | 0.1315 | 0.7890 |  |
| 8 | 85.19 (± 17.68) | 77.7 (± 12.86) | 0.3726 | 1.0000 |  |
| 9 | 86.32 (± 9.19) | 78.05 (± 11.01) | 0.3372 | 1.0000 |  |
| 10 | 92.34 (± 6.35) | 81.44 (± 11.20) | 0.2502 | 1.0000 |  |

| **ANOVA ON LME** |  |  |  |  |
| --- | --- | --- | --- | --- |
| Group | F(1,26) = 15.14, p = 0.0004 |  |  |  |
| Session | F(9,233) = 52.05, p = <.0001 |  |  |  |
| Group*Session | F(9,233) = 7.83, p = <.0001 |  |  |  |

**Supplementary Table 5.** Percentage of conditioned responses (CRs) in the 250 ms ISI training paradigm. All values represent mean ± 95% confidence interval.

|  | **CR percentage** | | **Comparisons** | | |
| --- | --- | --- | --- | --- | --- |
| **Day** | **Standard (n=12)** | **Enriched (n=16)** | **p-value** | **Bonf-Holm p-value** | |
| 1 | 71.21 (± 14.20) | 63.62 (± 13.16) | 0.5463 | 1.0000 |  |
| 2 | 79.44 (± 11.04) | 70.23 (± 15.49) | 0.3748 | 1.0000 |  |
| 3 | 76.39 (± 17.44) | 71.07 (± 11.93) | 0.5975 | 1.0000 |  |
| 4 | 73.05 (± 19.82) | 69.32 (± 11.62) | 0.7067 | 1.0000 |  |
| 5 | 88.21 (± 10.13) | 71.59 (± 14.72) | 0.1004 | 0.8032 |  |
| 6 | 68.99 (± 16.13) | 61.43 (± 12.50) | 0.5183 | 1.0000 |  |
| 7 | 87.10 (± 14.12) | 67.09 (± 15.07) | 0.0700 | 0.6680 |  |
| 8 | 78.43 (± 15.50) | 74.11 (± 14.77) | 0.7605 | 1.0000 |  |
| 9 | 84.16 (± 12.05) | 63.03 (± 18.04) | 0.0668 | 0.6680 |  |
| 10 | 76.77 (± 27.01) | 59.54 (± 17.21) | 0.1496 | 1.0000 |  |

| **ANOVA ON LME** |  |  |  |  |
| --- | --- | --- | --- | --- |
| Group | F(1,26) = 2.06, p = 0.1634 |  |  |  |
| Session | F(9,228) = 1.80, p = 0.0684 |  |  |  |
| Group*Session | F(9,228) = 0.78, p = 0.6386 |  |  |  |

**Supplementary Table 6.** Percentage of conditioned responses (CRs) in the 500 ms ISI training paradigm. All values represent mean ± 95% confidence interval.

|  | **Latency to CR onset** | | **Comparisons** | | |
| --- | --- | --- | --- | --- | --- |
| **Day** | **Standard (n=12)** | **Enriched (n=16)** | **p-value** | **Bonf-Holm p-value** | |
| 1 | 185.38 (± 119.31) | 259.03 (± 91.96) | 0.1696 | 1.0000 |  |
| 2 | 226.19 (± 97.88) | 259.20 (± 108.06) | 0.1452 | 1.0000 |  |
| 3 | 164.26 (± 29.18) | 286.60 (± 133.83) | 0.0003 | 0.0030 |  |
| 4 | 159.04 (± 18.80) | 246.02 (± 87.32) | 0.0139 | 0.1251 |  |
| 5 | 148.52 (± 13.76) | 175.50 (± 49.93) | 0.3442 | 1.0000 |  |
| 6 | 169.42 (± 35.21) | 170.74 (± 20.56) | 0.7468 | 1.0000 |  |
| 7 | 152.16 (± 24.46) | 184.46 (± 16.81) | 0.2497 | 1.0000 |  |
| 8 | 151.48 (± 25.53) | 169.63 (± 18.85) | 0.5102 | 1.0000 |  |
| 9 | 151.06 (± 22.35) | 164.33 (± 17.07) | 0.6488 | 1.0000 |  |
| 10 | 145.92 (± 33.40) | 161.43 (± 17.17) | 0.6525 | 1.0000 |  |

| **ANOVA ON LME** |  |  |  |  |
| --- | --- | --- | --- | --- |
| Group | F(1,26) = 7.23, p = 0.0123 |  |  |  |
| Session | F(9,196) = 2.76, p = 0.0047 |  |  |  |
| Group*Session | F(9,196) = 1.60, p = 0.1186 |  |  |  |

**Supplementary Table 7.** Latency to CR onset in the 250 ms ISI training paradigm. All values represent mean ± 95% confidence interval.

|  | **Latency to CR onset** | | **Comparisons** | | |
| --- | --- | --- | --- | --- | --- |
| **Day** | **Standard (n=12)** | **Enriched (n=16)** | **p-value** | **Bonf-Holm p-value** | |
| 1 | 142.50 (± 21.99) | 177.11 (± 30.66) | 0.1446 | 1.0000 |  |
| 2 | 132.90 (± 21.58) | 149.76 (± 26.47) | 0.4124 | 1.0000 |  |
| 3 | 141.07 (± 24.76) | 162.53 (± 22.05) | 0.2924 | 1.0000 |  |
| 4 | 140.42 (± 14.97) | 158.79 (± 24.26) | 0.3542 | 1.0000 |  |
| 5 | 149.52 (± 19.49) | 165.46 (± 38.05) | 0.4450 | 1.0000 |  |
| 6 | 174.22 (± 20.51) | 195.19 (± 52.74) | 0.3037 | 1.0000 |  |
| 7 | 174.15 (± 22.34) | 191.05 (± 33.14) | 0.3929 | 1.0000 |  |
| 8 | 156.24 (± 33.07) | 162.02 (± 40.18) | 0.7385 | 1.0000 |  |
| 9 | 176.72 (± 30.34) | 169.77 (± 33.15) | 0.8429 | 1.0000 |  |
| 10 | 188.36 (± 25.72) | 185.36 (± 31.31) | 0.9623 | 1.0000 |  |

| **ANOVA ON LME** |  |  |  |  |
| --- | --- | --- | --- | --- |
| Group | F(1,26) = 1.08, p = 0.3076 |  |  |  |
| Session | F(9,224) = 5.05, p = <.0001 |  |  |  |
| Group*Session | F(9,224) = 0.55, p = 0.8395 |  |  |  |

**Supplementary Table 8.** Latency to CR onset in the 500 ms ISI training paradigm. All values represent mean ± 95% confidence interval.

|  | **Latency to CR peak** | | **Comparisons** | | |
| --- | --- | --- | --- | --- | --- |
| **Day** | **Standard (n=12)** | **Enriched (n=16)** | **p-value** | **Bonf-Holm p-value** | |
| 1 | 448.60 (± 235.23) | 530.54 (± 140.86) | 0.3792 | 1.0000 |  |
| 2 | 399.06 (± 222.48) | 536.16 (± 203.06) | 0.0374 | 0.3366 |  |
| 3 | 338.61 (± 124.73) | 651.27 (± 349.27) | 0.0003 | 0.0030 |  |
| 4 | 302.18 (± 44.27) | 392.80 (± 123.84) | 0.1698 | 1.0000 |  |
| 5 | 283.54 (± 24.16) | 338.99 (± 80.16) | 0.3204 | 1.0000 |  |
| 6 | 317.23 (± 55.22) | 301.86 (± 63.86) | 0.9326 | 1.0000 |  |
| 7 | 309.32 (± 31.21) | 317.10 (± 48.87) | 0.8819 | 1.0000 |  |
| 8 | 320.63 (± 33.46) | 296.98 (± 42.54) | 0.6538 | 1.0000 |  |
| 9 | 313.66 (± 36.00) | 306.74 (± 45.18) | 0.9042 | 1.0000 |  |
| 10 | 325.84 (± 33.41) | 303.70 (± 49.79) | 0.7514 | 1.0000 |  |

| **ANOVA ON LME** |  |  |  |  |
| --- | --- | --- | --- | --- |
| Group | F(1,26) = 1.93, p = 0.1765 |  |  |  |
| Session | F(9,196) = 3.47, p = 0.0005 |  |  |  |
| Group*Session | F(9,196) = 2.00, p = 0.0418 |  |  |  |

**Supplementary Table 9.** Latency to CR peak in the 250 ms ISI training paradigm. All values represent mean ± 95% confidence interval.

|  | **Latency to CR peak** | | **Comparisons** | | |
| --- | --- | --- | --- | --- | --- |
| **Day** | **Standard (n=12)** | **Enriched (n=16)** | **p-value** | **Bonf-Holm p-value** | |
| 1 | 396.63 (± 83.67) | 377.43 (± 72.92) | 0.8180 | 1.0000 |  |
| 2 | 370.79 (± 60.16) | 361.14 (± 42.73) | 0.8501 | 1.0000 |  |
| 3 | 460.15 (± 108.86) | 412.81 (± 82.07) | 0.3479 | 1.0000 |  |
| 4 | 443.93 (± 97.96) | 426.95 (± 63.67) | 0.7323 | 1.0000 |  |
| 5 | 521.10 (± 87.80) | 459.00 (± 76.25) | 0.2239 | 1.0000 |  |
| 6 | 507.25 (± 58.38) | 527.91 (± 76.71) | 0.6874 | 1.0000 |  |
| 7 | 570.08 (± 41.08) | 506.97 (± 64.34) | 0.2215 | 1.0000 |  |
| 8 | 690.45 (± 67.44) | 574.97 (± 80.00) | 0.7549 | 1.0000 |  |
| 9 | 618.23 (± 55.39) | 538.23 (± 88.46) | 0.1260 | 1.0000 |  |
| 10 | 644.17 (± 67.7) | 544.50 (± 108.76) | 0.1596 | 1.0000 |  |

| **ANOVA ON LME** |  |  |  |  |
| --- | --- | --- | --- | --- |
| Group | F(1,26) = 0.79, p = 0.3810 |  |  |  |
| Session | F(9,224) = 10.95, p = <.0001 |  |  |  |
| Group*Session | F(9,224) = 0.76, p = 0.6514 |  |  |  |

**Supplementary Table 10.** Latency to CR peak in the 500 ms ISI training paradigm. All values represent mean ± 95% confidence interval.

|  | **Perfect CR (%)** | | **Comparisons** | | |
| --- | --- | --- | --- | --- | --- |
| **Day** | **Standard (n=12)** | **Enriched (n=16)** | **p-value** | **Bonf-Holm p-value** | |
| 7-10 | 39.39 (± 4.47) | 57.56 (± 4.17) |  |  |  |

| **ANOVA ON LME** |  |  |  |  |
| --- | --- | --- | --- | --- |
| Group | F(1,26) = 5.30, p = 0.0296 |  |  |  |

**Supplementary Table 11.** Perfectly timed window of CR percentage between in the 250 ms ISI training paradigm. All values represent mean ± 95% confidence interval.

|  | **Perfect CR (%)** | | **Comparisons** | | |
| --- | --- | --- | --- | --- | --- |
| **Day** | **Standard (n=12)** | **Enriched (n=16)** | **p-value** | **Bonf-Holm p-value** | |
| 17-20 | 28.69 (± 4.66) | 35.92 (± 4.65) |  |  |  |

| **ANOVA ON LME** |  |  |  |  |
| --- | --- | --- | --- | --- |
| Group | F(1,25) = 1.31, p = 0.2630 |  |  |  |

**Supplementary Table 12.** Perfectly timed window of CR percentage between in the 500 ms ISI training paradigm. All values represent mean ± 95% confidence interval.

|  | **Standard (n = 11)** | **Enriched (n = 16)** | **p-value** | **Bonf-Holm p-value** |
| --- | --- | --- | --- | --- |
| **Balance beam** |  |  |  |  |
| Time to cross 6-mm beam (s) | 8.70 (±1.65) | 7.83 (±1.58) |  |  |
| Time to cross 12-mm beam (s) | 6.48 (±1.67) | 6.03 (±1.57) |  |  |
| **ANOVA on LME** |  |  |  |  |
| Group | F(1,25) = 0.03, p = 0.8654 |  |  |  |
| Beam | F(1,77) = 4.54, p = 0.0362 |  |  |  |
| Group*Beam | F(1,77) = 0.82, p = 0.3671 |  |  |  |
|  |  |  |  |  |
| **Grip strength test** |  |  |  |  |
| Grip strength (N) | 90.00 (±4.11) | 102.00 (±4.62) |  |  |
| **ANOVA on LME** |  |  |  |  |
| Group | F(1,25) = 2.48, p = 0.1281 |  |  |  |
|  |  |  |  |  |
| **Accelerating Rotarod** |  |  |  |  |
| Day 1 - Latency to fall (s.) | 108.18 (±48.08) | 192.25 (±33.14) | p=0.0003 | p=0.0010 |
| Day 2 - Latency to fall (s.) | 159.89 (±42.72) | 226.09 (±31.25) | p=0.0026 | p=0.0077 |
| Day 3 - Latency to fall (s.) | 179.61 (±45.36) | 222.15 (±28.34) | p=0.0411 | p=0.0821 |
| Day 4 - Latency to fall (s.) | 178.68 (±47.04) | 213.13 (±34.21) | p=0.0934 | p=0.0934 |
| **ANOVA on LME** |  |  |  |  |
| Group | F(1,25) = 9.72, p = 0.0046 |  |  |  |
| Day | F(1,399) = 25.28, p = <.0001 |  |  |  |
| Group*Day | F(1,399) = 6.65, p = 0.0002 |  |  |  |

**Supplementary Table 13.** Outcome measures motor performance during the balance beam test, grip strength test, and the accelerating rotarod. All values represent median ± 95% confidence interval, for both balance beam, and grip strength test. All accelerating rotarod values represent mean ± 95% confidence interval.

| **Mouse** | **Day 1** | **Day 2** | **Day 3** | **Day 4** | **Day 5** |
| --- | --- | --- | --- | --- | --- |
| 2710 | 57.53(±3.01) | 62.16(±2.67) | 61.08(±3.25) | 60.63(±2.69) | 59.99(±3.80) |
| 2711 | 56.11(±2.91) | 37.28(±2.51) | 63.49(±2.28) | 66.80(±1.90) | 66.71(±1.96) |
| 2714 | 46.52(±3.59) | 40.80(±4.00) | 46.88(±3.48) | 51.22(±3.41) | 52.11(±2.51) |
| 2717 | 61.02(±3.45) | 64.77(±3.56) | 62.16(±2.77) | 63.59(±2.25) | 66.14(±2.50) |
| 2721 | 61.41(±4.16) | 66.37(±3.24) | 63.32(±2.26) | 61.75(±3.66) | 61.11(±2.88) |
| 2723 | 72.35(±4.15) | 69.01(±2.55) | 64.11(±2.24) | 66.76(±1.57) | 67.22(±1.90) |
| 2725 | 46.21(±4.53) | 48.61(±3.72) | 45.45(±3.41) | 51.89(±4.21) | 52.75(±4.18) |
| 2727 | 49.82(±3.88) | 53.14(±3.72) | 51.22(±3.37) | 56.82(±3.07) | 53.50(±3.13) |
| 2817 | 70.23(±4.47) | 82.68(±3.82) | 81.16(±3.94) | 84.99(±2.21) | 86.67(±3.75) |
| 2818 | 69.58(±4.39) | 72.62(±3.90) | 75.31(±3.94) | 75.80(±3.63) | 74.57(±3.15) |
| 2819 | 71.13(±4.40) | 81.00(±5.34) | 81.59(±4.36) | 85.69(±2.48) | 80.33(±3.74) |
| 2821 | 65.13(±5.35) | 71.26(±4.50) | 74.27(±3.63) | 74.38(±3.51) | 69.28(±5.07) |
| 2822 | 74.02(±4.94) | 85.13(±3.62) | 84.93(±2.67) | 88.02(±2.60) | 74.71(±4.77) |
| 2823 | 69.35(±5.26) | 71.18(±4.21) | 75.29(±4.65) | 75.55(±4.74) | 68.61(±3.80) |
| 2826 | 71.51(±3.93) | 81.35(±3.29) | 84.54(±2.83) | 87.14(±3.59) | 83.91(±3.41) |
| 2827 | 66.60(±3.80) | 77.53(±2.84) | 75.54(±2.71) | 75.78(±3.58) | 74.54(±4.83) |

**Supplementary Table 14.** Percentage of correct steps per individual enriched-housed mouse. All values represent mean ± 95% confidence interval.

| **Mouse** | **Day 1** | **Day 2** | **Day 3** | **Day 4** | **Day 5** |
| --- | --- | --- | --- | --- | --- |
| 2713 | 46.38(±3.79) | 57.28(±4.24) | 60.71(±3.38) | 64.99(±2.67) | 67.52(±3.08) |
| 2715 | 39.54(±4.31) | 54.90(±3.98) | 56.49(±3.34) | 60.17(±3.18) | 58.98(±3.20) |
| 2716 | 34.17(±3.65) | 45.59(±3.55) | 51.02(±3.33) | 51.75(±3.49) | 54.78(±3.29) |
| 2722 | 62.06(±5.10) | 65.92(±3.88) | 59.92(±2.32) | 61.89(±1.82) | 62.14(±2.64) |
| 2724 | 46.02(±4.56) | 53.45(±4.02) | 55.33(±3.02) | 54.51(±3.16) | 60.08(±2.69) |
| 2726 | 40.76(±3.76) | 52.62(±3.64) | 56.05(±2.76) | 55.31(±3.35) | 58.01(±2.76) |
| 2728 | 54.44(±4.50) | 62.44(±3.90) | 55.56(±4.08) | 58.35(±3.18) | 59.96(±2.80) |
| 2816 | 63.88(±4.50) | 75.95(±3.10) | 79.54(±2.71) | 77.33(±4.61) | 80.06(±3.40) |
| 2820 | 59.04(±4.31) | 73.54(±5.20) | 73.44(±4.67) | 79.89(±3.15) | 72.73(±4.99) |
| 2824 | 57.46(±5.02) | 68.72(±5.91) | 69.67(±4.55) | 70.32(±5.17) | 72.28(±3.98) |
| 2825 | 49.23(±3.91) | 58.30(±4.28) | 60.34(±4.52) | 62.97(±4.27) | 60.96(±3.87) |

**Supplementary Table 15.** Percentage of correct steps per individual standard-housed mouse. All values represent mean ± 95% confidence interval.

|  | **Standard (n = 11)** | **Enriched (n = 16)** | **p-value** | **Bonf-Holm p-value** |
| --- | --- | --- | --- | --- |
| **ErasmusLadder** |  |  |  |  |
| Day 1 - Correct steps (%) | 50.27 (±4.31) | 63.03 (±4.14) | p = 0.0054 | p = 0.0268 |
| Day 2 - Correct steps (%) | 60.79 (±4.15) | 66.55 (±3.59) | p = 0.1175 | p = 0.3871 |
| Day 3 - Correct steps (%) | 61.63 (±3.52) | 68.15 (±3.24) | p = 0.1206 | p = 0.3871 |
| Day 4 - Correct steps (%) | 63.41 (±3.46) | 70.43 (±3.07) | p = 0.0968 | p = 0.3871 |
| Day 5 - Correct steps (%) | 64.32 (±3.34) | 68.26 (±3.46) | p = 0.3530 | p = 0.3871 |
|  |  |  |  |  |
| **ANOVA on LME** |  |  |  |  |
| Group | F(1,25) = 3.26. p = 0.0833 |  |  |  |
| Day | F(4,5376) = 110.99. p = <.0001 |  |  |  |
| Group*Day | F(4,5376) = 17.92. p = <.0001 |  |  |  |

**Supplementary Table 16.** Percentage of correct steps for standard-housed and enriched-housed mice. All values represent mean ± 95% confidence interval.
